# The Leicester Royal Infirmary

**Published:** 1916-05-27

**Authors:** 


### THE LEICESTER ROYAL INFIRMARY.

### A New Chairman Appointed.

At the monthly meeting of the Board of Governors of the Leicester Royal Infirmary, Mr. T. Fielding Johnson, jun., J.P., was unanimously elected Chairman of the Infirmary for a period of three years. It will be remembered that the board adopted the plan of terminal appointments recently in the cases of the Treasurer and the Chaplains, the principle being that interest in the institution would be increased rather than diminished by appointments for a limited period. Mr. Fielding Johnson's appointment is of additional interest from the fact that his father, Mr. T. Fielding Johnson, who still survives, and is keenly interested in the institution, is the oldest governor, and was, during the years 1898 to 1902, Chairman of the institution. Mr. Fielding Johnson, jun., has rendered great service for many years as Chairman of the House Committee (the Administrative Committee), and as a trustee of the institution. His unanimous election to the chairmanship of the board is happy testimony to the ability, urbanity, and unvarying courtesy with which he has discharged his duties as Chairman of this Committee. During this time a great reconstruction scheme has been carried out and the equipment thoroughly reorganised at a cost of £120,000. Colonel C. J. Bond, the Vice-Chairman of the board, who has acted as Chairman since Mr. Simpson Gee's resignation of the office, will continue as Vice-Chairman. Leicester is fortunate in securing a thoroughly experienced gentleman as its Chairman, who has already shown marked ability and tact in dealing with the difficulties which occasionally present themselves in hospital administration.

### The Financial Position Encouraging.

It was reported at the meeting that through the energy of the House Governor and Secretary, the debt on the Children's Hospital of £2,050 had been liquidated, through a challenge issued by the Mayor of Leicester, Alderman Jonathan North, J.P., to contribute £250 from his firm if the debt were liquidated in a month. This condition had been readily met, and in addition a sum of £300 had been provided for the furnishing and equipment of the operating theatre and ante-rooms attached to the Children's Hospital. Considerable progress had been made with the raising of funds for the construction of a new pathological laboratory and a new mortuary viewing room and chapel, estimated to cost £4,500 to £5,000. In addition to £1,000 promised by Sir Edward Wood, £500 by Mr. Simpson Gee, and £100 by Mr. Fielding Johnson, sen., all former Chairmen of the board, and £100 each by the new Chairman and Deputy-Chairman (£1,800 in all), £1,200 had been promised in fresh contributions, making a total of £3,000 toward the amount required. Through the instrumentality of Mr. B. W. Russell, the Chairman of the Finance Committee, a sum of about £450 in new and increased annual subscriptions was announced. Mr. and Mrs. Robert Hyslop, son-in-law and daughter of Sir Edward Wood, have sent a cheque for £1,000 for the permanent endowment of two cots in the Children's Hospital, to the memory of their two daughters, Miss Mary and Miss Annie Louise Hyslop. A legacy of £100 from Mr. T. J. Thorneloe was also reported. The institution thus commences a new era of service with an encouraging financial outlook.

